# Blood pressure measurement and nocturnal dipping patterns are heavily affected by body posture through changes in hydrostatic pressure between the arm and the heart

**DOI:** 10.1038/s41440-024-02056-0

**Published:** 2024-12-06

**Authors:** Niklas Pilz, Krzysztof Narkiewicz, Jacek Wolf, Kazuomi Kario, Tinta Visser, Oliver S. Opatz, Alma Reuter, Laura J. Dippel, Leon Fesseler, Viktor Heinz, Andreas Patzak, Tomas L. Bothe

**Affiliations:** 1 Charité—Universitätsmedizin Berlin, Institute of Physiology, Center for Space Medicine and Extreme Environments Berlin, Berlin, Germany; 2 Department of Cardiology and Angiology, Hannover Medical School, Hannover, Germany; 3 Department of Hypertension and Diabetology, Medical University of Gdansk, Gdansk, Poland; 4 Division of Cardiovascular Medicine, Department of Medicine, Jichi Medical University School of Medicine, Tochigi, Japan; 5 SOMNOmedics GmbH, Randersacker, Germany; 6 Charité—Universitätsmedizin Berlin, Institute of Translational Physiology, Berlin, Germany

**Keywords:** Blood pressure monitoring, Ambulatory, Circadian rhythm, Posture, Hydrostatic pressure, Hypertension

## Abstract

Nocturnal blood pressure (BP) shows the highest predictive power for cardiovascular events. However, there is a poor reproducibility of personalized dipping patterns in single individuals. We hypothesize that changes in body position during sleep cause variations in hydrostatic pressure,leading to incorrect BP values and dipping classifications. 26 subjects aged 18–30 years, as well as 25 participants aged 50 years and older underwent ambulatory BP measurements on the left arm, as well as determination of the hydrostatic pressure difference between the cuff and heart level during BP measurement. We observed that the BP measurement cuff was above the heart level (negative hydrostatic pressure) mostly through the night. Laying on the right side revealed the largest hydrostatic pressure difference and maximum incorrect BP measurement, with a mean of –9.61 mmHg during sleep. Correcting for hydrostatic pressure led to reclassification of nocturnal hypertension in 14 subjects (27.5%). Dipping patterns changed in 19 participants (37.3%). In total, 25 subjects (49.0%) changed either their nocturnal hypertension and/or their dipping classification. Our findings underscore the importance of accounting for hydrostatic pressure in ambulatory BP monitoring. Changes in body posture during sleep provide a plausible reason for the variability seen in nocturnal dipping patterns. Further research should focus on incorporating hydrostatic pressure compensation mechanisms in 24-h BP measurement. Limiting the noticeable effect of hydrostatic pressure differences could greatly improve hypertension diagnosis, classification, and treatment monitoring.

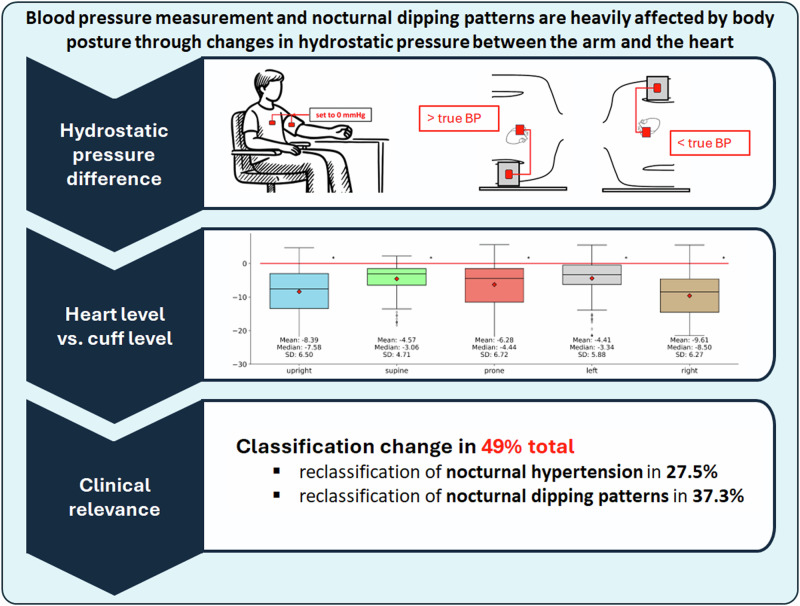

## Introduction

Arterial hypertension constitutes a crucial cardiovascular risk factor with a widespread presence in everyday medical practice. It contributes to various cardiovascular diseases and strongly impacts overall health and well-being [1–6]. Accurate blood pressure (BP) monitoring is important for reliable diagnosis and effective management of hypertension [7].

Ambulatory BP monitoring (ABPM) is considered the gold standard for hypertension diagnosis, as it provides BP measurements throughout the day, including nocturnal BP. The assessment of nocturnal BP offers additional insights into cardiovascular health: Nocturnal hypertension and pathological dipping patterns, such as non-dipping and reverse dipping, are the strongest blood pressure related predictors of cardiovascular risk [8–12]. In consequence, establishing a dependable diagnostic framework for nocturnal BP measurements is of paramount importance for improving cardiovascular risk prediction and therefore greatly impacts clinical decision-making [13, 14].

However, although the clinical value of ABPM is well established especially nocturnal BP measurements suffer from poor reproducibility [8, 12, 15]. Several factors contribute to the challenges in obtaining consistent results: Cuff-based, ABPM is prone to measurement artefacts [15, 16]. Further, the diversity in sleep patterns among patients introduces variability in time-frame and duration of sleep [17]. This affects the patient-specific nighttime period and therefore the ideal patient-specific nocturnal BP detection [16–18].

According to the guidelines, BP should be measured at heart level [7, 19, 20]. The reason for that is the influence of the hydrostatic pressure. BP values decrease towards the head and increase towards the feet by 0.735 mmHg/cm in upright position. The same hydrostatic effects occur when patients with a pressure cuff on one arm lie on the left or right side [21]. As measurement with the arm on heart level is the gold standard, BP is too low when the cuff is above the heart and vice versa. When in a side position, the maximum height difference between the cuff on the upper arm and the heart level can exceed 20 cm. Patients change body postures during an ABPM. Particularly during the night, natural movements of patients result in a deviation from the intended cuff and body position [22, 23].

This study aims to investigate the influence of hydrostatic pressure differences between the heart and the BP cuff on nocturnal BP in ABPM, with the goal of further improving its accuracy. We hypothesize that the effect of hydrostatic pressure leads to incorrect BP measurement and classification, and wrong determination of dipping patterns. In more detail, we compare the effect in two subgroups: Young and healthy adults aged 18–30 and a group of subjects aged 50 years and older. This was done to compare the sole physiological effect size to the impact of hydrostatic pressure on a more realistic patient subsample. Finally, it is our goal to evaluate the feasibility and the clinical effect of correcting hydrostatic pressure differences.

## Methods

### Participants

Our study encompassed two distinct subgroups: a group of young and healthy subjects ranging from 18 to 30 years of age (younger), and a cohort comprising older participants aged 50 years and above (older). The sole exclusion criterion for the younger subgroup was the presence of diagnosed cardiovascular diseases. No further exclusion criteria were applied in this study.

### Set-up and devices

To perform oscillometric BP measurements, we attached a BoSo TM-2430 device (BOSCH + SOHN GmbH u. Co. KG, Jungingen, Germany; outside of Germany: *A&D TM-2430*, A&D Inc., Tokyo, Japan) to the subjects’ left arm. The cuff sizes were selected appropriate for individual arm dimensions as recommended by the manufacturer [24].

To determine the hydrostatic pressure difference between the heart level and the cuff, we used the height correction unit of the Finometer® Midi (Finapres Medical Systems B.V., Enschede, Netherlands). The sensor was disconnetected from the Finometer® and connected to our central data recorder [25, 26]. The unit was calibrated and tested on defined height differences. It showed a measurement error of below 1 mmHg within laboratory conditions for a range of ±30 mmHg.

**Fig. 1:**
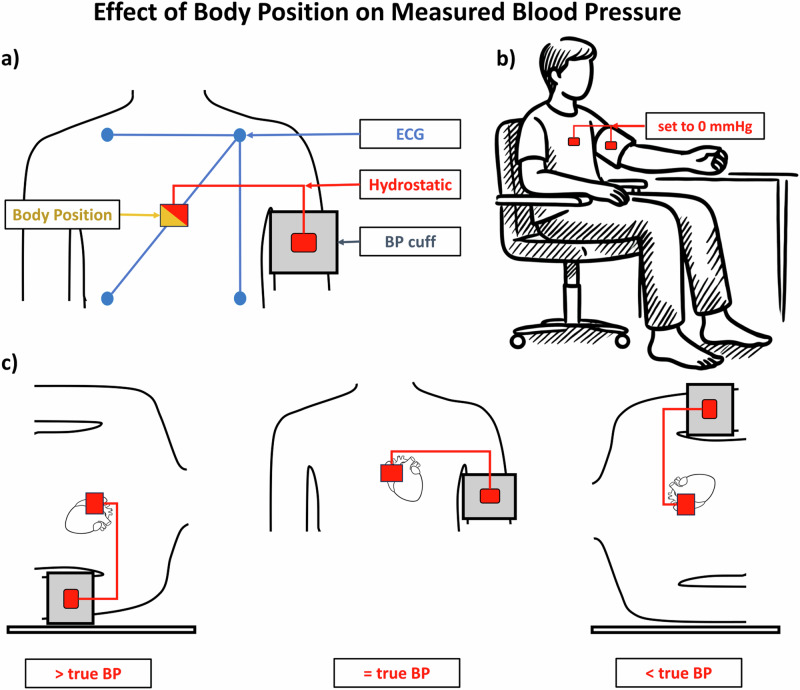
Effect of body position on measured blood pressure: The upper-left panel (**a**) depicts the measurement setup. The ECG (blue), BP (grey), body position (yellow), and hydrostatic pressure (red) sensors are shown. The upper-right panel shows the recommended position for BP measurement with cuff at the heart level. We calibrated the measured hydrostatic pressure in that position to 0 mmHg (upper right panel **b**). The lower panel (**c**) shows examples of the effect of changes in body position on the measured blood pressure. The measured BP is lower than the true (heart level) BP when the arm is above the heart level and higher when below

We used a SOMNOtouch NIBP™ (SOMNOmedics, Randersacker, Germany) as data recorder [27, 28]. This device enabled us to record theECG and the central-thoracal body position in axial positions. Further, we used a pressure transducer and a Y-connection to the pressure hose to record the pressure within the TM-2430’s BP cuff. This method has been reliably used in earlier studies [15, 16] (Fig. 1).

### Procedure

After attachment of all devices, we initiated the SOMNOtouch NIBP™ (SOMNOmedics GmbH, Randersacker, Germany) and checked the signal quality of all recorded traces.

Following this, we conducted a test BP measurement with the BoSo TM-2430. This measurement was taken in accordance with the recommendations for BP measurement, with seated patients and with arms rested at heart level. The hydrostatic pressure difference between the arm and the aortic valve level was set as zero difference. This was done as the BP in a seated position is considered the gold-standard for BP measurement and guidelines recommend performing measurement like this [7]. All subsequently recorded hydrostatic pressure differences could therefore be interpreted as difference from the position recommended by the guidelines. A negative value therefore corresponds to the cuff being above the heart level and vice versa (Fig. 1).

We then switched to the automatic ABPM mode. In accordance with recommendations for ABPM at the start of the data collection phase, BP was measured at fifteen-minute intervals between 7 a.m. and 10 p.m. and at half-hour intervals between 10 p.m. and 7 a.m.

We connected the measurement devices at least one hour before bedtime and removed them no earlier than one hour after awakening.

Participants were asked to report their sleep times as precise as possible. BP measurements conducted during this period were classified as nocturnal. An upright position during the night might occur during situations such as using the toilet or briefly sitting up. More generally, subjects sleeping in a seated or inclined position.

### Data processing and statistical analysis

Initially, we screened the BP measurements for artefacts. Following the recommendations outlined in current research, any BP readings that exhibited a rise in cuff pressure of more than 8 mmHg during deflation were excluded from further analysis [15, 16].

We continuously recorded and analysed the hydrostatic pressure differences between the cuff level and heart level throughout the entire measuring period. This included comparisons between both subgroups as well as between measurements taken while being asleep and awake. The hydrostatic pressure difference between heart level and cuff level was averaged above each single BP measurement, which allowed us to obtain a specific value for the hydrostatic pressure difference for each single BP reading. Thus, we were able to correct every single BP measurement based on the corresponding hydrostatic pressure at the time of measurement. Additionally, we investigated the hydrostatic pressure differences in different body positions. Furthermore, we examined the clinical relevance in terms of changes in classification of nocturnal hypertension and dipping patterns for individual subjects when correcting for hydrostatic pressure.

Lastly, we evaluated whether correcting the measured blood pressure using the mean hydrostatic pressure difference for each axial body position could meaningfully reduce the error induced by changes in body position. This was done as determining the current body position could be more easily incorporated in everyday ABPM measurement without the need of a direct measurement of the hydrostatic pressure difference. The results of this analysis are presented in a supplemental file. (Supplement 1)

We conducted all analyses using Python 3.9 and the SciPy statistics library [29]. Differences in mean were analysed by the appropriate paired or unpaired t-tests or ANOVA with Tukey’s HSD post hoc test. The alpha-level was set at *p* < 0.05.

### Ethics

The study was approved by the local ethics committee (ethics committee Campus Charité Mitte, Charité—Universitätsmedizin Berlin, approval-number: EA4/278/21). The study was registered at Charité—Universitätsmedizin Berlin’s clinical trial register before the start of data collection (ePA: 3000457). All subjects gave their written and informed consent to partake in this study.

## Results

We conducted the study in 51 subjects. On average single measurement lasted 12:48 h (SD: 146 min) and included 34 BP measurements (SD: 8) Overall, 1,715 BP measurements with complete data recording were retrieved. 981 measurements (57.2%) where recorded awake and 734 (42.8%) asleep. (Table 1).

**Table 1:** Dataset composition

	Total (*N* = 51)	younger (*N* = 26)	Older (*N* = 25)	Male (*N* = 24)	Female (*N* = 27)
	Mean ± SD	Mean ± SD	Mean ± SD	Mean ± SD	Mean ± SD
**Age in years**	44.2 ± 23.6	22.3 ± 2.3	66.9 ± 9.9	45.3 ± 23.7	43.1 ± 23.9
**Height in cm**	174.8 ± 12.0	176.7 ± 13.0	172.8 ± 10.8	184.2 ± 9.5	166.5 ± 6.7
**Weight in kg**	73.9 ± 16.2	71.3 ± 16.4	76.6 ± 15.8	84.2 ± 12.9	71.3 ± 16.4
**BMI in kg / m**^**2**^	24.1 ± 4.2	22.5 ± 2.4	25.7 ± 5.1	24.7 ± 2.5	23.5 ± 5.3
**Mean SBP in mmHg**	121.5 ± 17.9	113.3 ± 11.2	129.8 ± 19.8	125.1 ± 9.9	118.0 ± 22.5
**Mean DBP in mmHg**	69.9 ± 7.6	65.5 ± 3.8	74.4 ± 8.0	70.7 ± 6.4	69.2 ± 8.6

*BMI* Body Mass Index, *SBP* systolic blood pressure, *DBP* diastolic blood pressure

After exclusion of 262 (15.0%) incorrect measurements, a total of 1453 (85.0%) measurements were not affected by artefacts in cuff pressure curves and therefore used for analysis. For the entire dataset, we excluded 206 (21.0%) of the awake measurements and 56 (7.6%) of the asleep measurements. In the younger cohort, 102 (18.9%) of measurements while being awake and 45 (11.6%) of measurements while being asleep were excluded. In the older subgroup, 104 (23.6%) of the awake measurements and 11 (3.2%) of the asleep measurements were removed.

### Hydrostatic pressure difference between cuff and heart level

We analysed the presence of hydrostatic pressure differences between the cuff and heart level during BP measurement. Both younger and older participants exhibited a substantial range of hydrostatic pressure differences. The average hydrostatic pressure difference per measurement between the heart and the cuff was higher in the younger subgroup (*p* < 0.001).

**Fig. 2:**
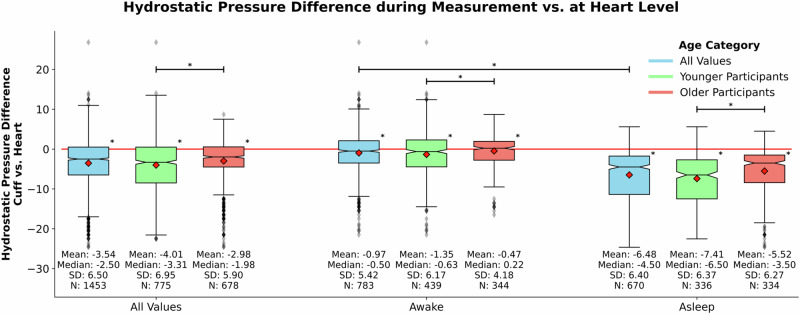
Hydrostatic pressure difference during measurement vs. at Heart Level: This figure delineates the deviation in hydrostatic pressure between the cuff and the heart level during BP measurement. We compared the hydrostatic pressure difference over the whole measurement as well as while awake and asleep. Distinctions are depicted between the whole data set, the younger (18–30 years) and older subgroup (50 years and above). Asterisks next to boxed indicate a significant effect compared to zero. Asterisks on top horizontal bars indicate differences between groups. * = *p* < 0.05

The most pronounced pressure differences occurred during the night with an average of –6.48 mmHg (indicating the cuff position to be above the heart level). Across the entire dataset, we noted a larger effect of hydrostatic pressure in measurements taken during sleep as opposed to those recorded while being awake (*p* < 0.001). (Fig. 2).

### Hydrostatic pressure difference depending on axial body posture

**Fig. 3:**
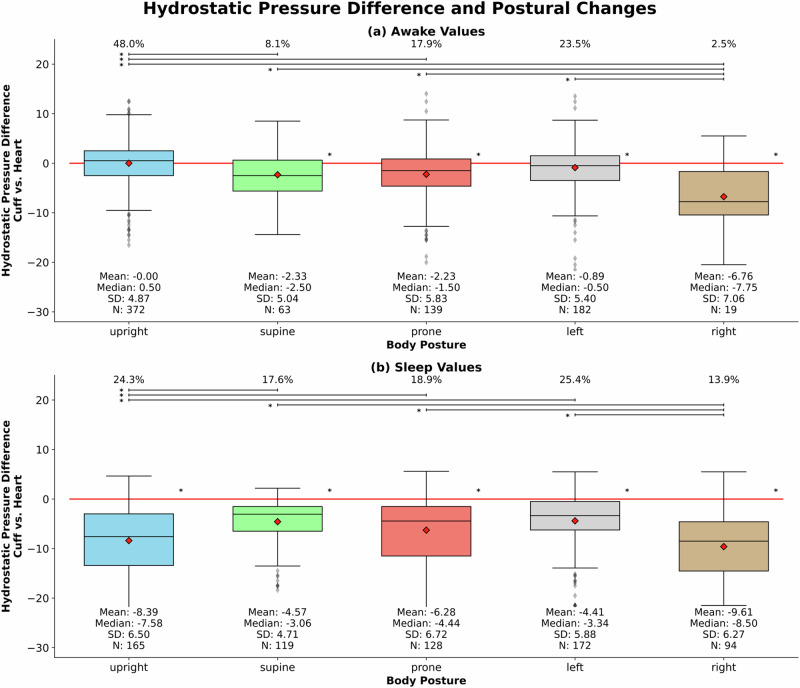
Hydrostatic pressure difference and postural changes: Boxplots represent hydrostatic pressure differences at cuff versus heart level for various axial body postures, stratified by awake state (**a**), and sleep state (**b**). * = *p* < 0.05, SD standard deviation

Analysing axial body postures revealed that the right-sided posture during sleep manifested the highest hydrostatic pressure difference with a mean of –9.61 mmHg. For all values and during the night, all body postures led to negative mean hydrostatic pressure differences, indicating the cuff being above the heart level. This is also true for daytime measurements aside from measurements taken in an upright position. Further, the data indicated mean differences between the distinct axial positions, especially pronounced during night (Fig. 3).

### Clinical relevance of hydrostatic pressure differences

**Fig. 4:**
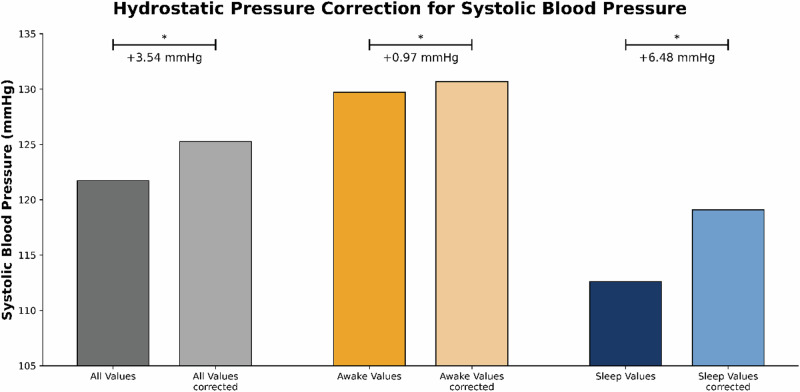
Systolic blood pressure and hydrostatic pressure difference correction: The figure depicts the comparison of mean systolic blood pressure values for all measurements compared to hydrostatic corrected values. Asterisks (*) indicate a statistical significance of *p* < 0.005

Correcting for hydrostatic pressure differences led to higher BP compared to uncorrected BP values (*p* < 0.005). The nocturnal values in particular showed a pronounced increase after correction, with a mean augmentation of 6.48 mmHg (*p* < 0.001) (Fig. 4).

**Fig. 5:**
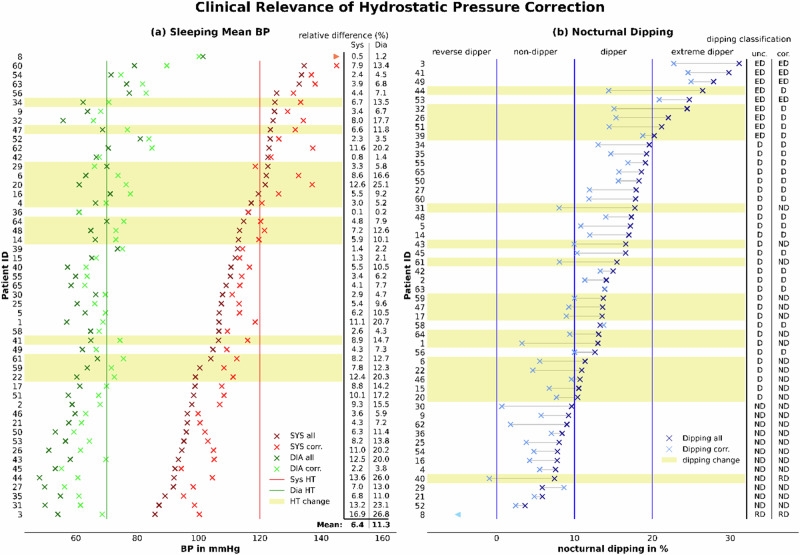
Effect of correcting nocturnal hydrostatic pressure differences on clinical classification: the figure depicts changes of nocturnal hypertension (**a**) and dipping pattern (**b**) classifications. A yellow background indicates a classification change in the respective patient. SYS systolic blood pressure, corr. corrected, DIA diastolic blood pressure, HT Hypertension, unc. uncorrected, cor. corrected, RD reverse dipper, ND non dipper, D dipper, ED extreme dipper

Our study revealed that correcting the hydrostatic pressure difference led to the reclassification of nocturnal hypertension across 14 (27.5%) subjects. Moreover, the correction for hydrostatic pressure resulted in changes in classification of nocturnal dipping patterns in 19 (37.3%) participants. Specifically, one individual’s status altered from a *non-dipper* to a *reverse dipper*. In 13 subjects the categorization changed from *dipper* to *non-dipper*, reflecting a diminished nocturnal BP decline. Additionally, five subjects were reclassified from an *extreme dipper* to *dipper*. Overall, 49.0% of participants (*N* = 25) changed either their nocturnal hypertension and/or their dipping classification (Fig. 5). Of the 25 affected patients, 14 were from the younger age group (14/26, 53.8%) and 11 were from the older age group (11/25, 44.0%).

**Fig. 6:**
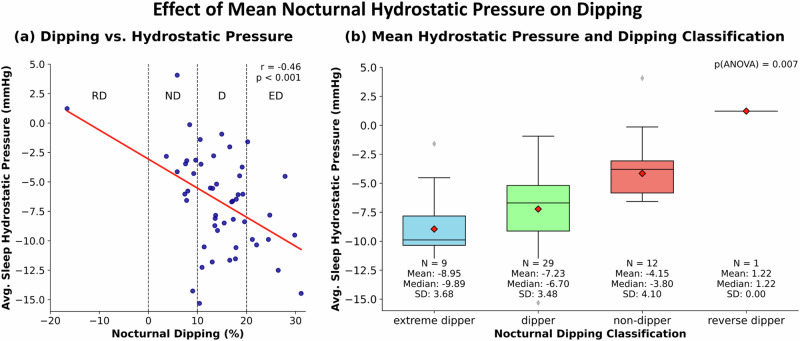
Effect of Mean Nocturnal Hydrostatic Pressure on Dipping: The left panel (**a**) depicts the connection between the mean nocturnal hydrostatic pressure difference between the cuff and the heart and the nocturnal dipping. The right panel (**b**) shows the difference in mean hydrostatic pressure difference between the four different dipping pattern classifications. RD reverse dipper, ND non dipper, D dipper, ED extreme dipper, Avg. average, N number of samples, SD standard deviation

Analysis of the mean nocturnal hydrostatic pressure differences of individual patients showed a correlation to the dipping pattern classification (*p* < 0.001). The negative correlation coefficient (r = –0.46) obtained signifies that an increased negative pressure difference (cuff above heart level) is connected to stronger dipping and vice versa. Accordingly, an ANOVA analysis revealed differences in mean nocturnal hydrostatic pressure for patients with different dipping classifications (*p* = 0.007). (Fig. 6)

### Feasibility of body posture derived correction of hydrostatic pressure differences

Lastly, we analysed the possibility of limiting the measurement error induced by not directly measuring the hydrostatic pressure difference. Correcting the hydrostatic pressure difference by using axial body postures reduced the absolute error for all body postures except from upright and left posture for all measurements and for measurements during the day [30] (*p* < 0.05). Moreover, it reduced the absolute error for all body postures during the night (*p* < 0.05). Especially for the right-side body posture, the absolute error was reduced by more than 50% during day and night. (Supplementary 1).

## Discussion

In this study, we established that hydrostatic pressure differences between the cuff and heart level strongly influence nocturnal BP measurement. This influence leads directly to clinically relevant changes in hypertension (27.4% of patients) and dipping pattern (37.2% of patients) classification. Overall, almost every second patient (49.0%) was affected by a change in clinical classification.

Generally, younger participants exhibited slightly larger absolute hydrostatic pressure differences compared to their older counterparts, both during awake and asleep periods. This difference may be attributed to the greater physical flexibility and increased mobility of younger individuals, allowing them to adopt a wider variety of positions throughout the day. This issue potentially contributes to more pronounced shifts in hydrostatic pressure. In contrast, older subjects may experience more physical restrictions due to age-related factors such as joint stiffness or musculoskeletal issues, which may limit their ability to change positions as frequently or comfortably. These limitations are likely to persist during both waking hours and sleep [31, 32]. However, differences between the age groups were marginal, greatly outsized by the common effect in both groups. This suggests that hydrostatic effects are relevant in all age groups.

Notably, especially the mean nocturnal BP rose by correcting the pressure difference, showcasing that the participants arm was above their heart level in most cases during the night. Moreover, the amplitude of the patient specific mean nocturnal hydrostatic pressure difference meaningfully correlated with the dipping pattern classification. This means that the retrieved dipping pattern from ABPM is, at least in part, not an effect of true, intrinsic changes of patient BP but rather an effect of a patient’s body position during sleep.

As laying on a repeatedly inflating BP cuff can be uncomfortable during the night, it is plausible that patients will predominantly position their arm, and therefore the BP cuff, above their thorax [30]. Our analysis revealed the largest hydrostatic pressure difference between heart level and cuff when patients were laying on their right side, placing the cuff on the left arm above the heart. The absolute hydrostatic pressure difference was less pronounced when subjects lay on their left side, which we attribute to the common tendency for subjects to lie on their flank instead of laying direct on their arm [33, 34].

Our findings emphasize the practical challenges measuring BP during sleep, given the guideline’s approach of ideally measuring the BP at heart level as closely as possible [35]. ABPM is considered the gold-standard in detecting nocturnal BP and dipping patterns [7, 36]. However, the ideal BP measurement conditions are particularly challenging to adhere for ABPM, especially during the night [7, 16, 20].

Given these study’s results, we hypothesize that hydrostatic pressure differences contribute to the limited reproducibility of dipping patterns, which is frequently reported in scientific literature [37–39]. Further, as correcting the pressure differences leads to an increase in mean BP, it could explain in part why the clinically cut-off thresholds are lower in ABPM than compared to office BP measurement [7].

To further enhance the predictive capability of ABPM, we suggest incorporating hydrostatic pressure differences into (nocturnal) BP interpretation. Hydrostatic pressure is an important influencing factor that affects BP measurements, particularly when changing body position. By accounting for these variations, the prognostic value of ABPM as the gold standard for hypertension diagnosis could be improved. Therefore, usage of hydrostatic corrected ABPM could lead to more accurate and reliable assessments of cardiovascular risk. Hydrostatic pressure can be directly measured, as demonstrated in this study. Further, our results demonstrate that correcting the hydrostatic pressure by using surrogate parameters such as the axial body position can greatly reduce its effect and therefore meaningfully improve BP measurement.

One of the main criticisms of cuff-less continuous BP monitoring is that these devices show limited nocturnal reduction of BP when comparing to ABPM [28, 40, 41]. Our findings reveal that nocturnal ABPM values are lower, when not corrected for hydrostatic differences, increasing the apparent nocturnal dipping. Accordingly, adjusting for hydrostatic differences could reduce the discrepancy between cuff-less and cuff-based devices in measuring nocturnal BP.

There are limitations to the generalizability of our study: Due to the methodological sophistication, we were only able to conduct our experiment in a limited number of patients. It is plausible that the effect of hydrostatic pressure is different in specific subgroups such as obstructive sleep apnoea patients, obese patients, pregnant women, children, etc. Further, our analysis assumed that the physical pressure difference is closely translated in a BP difference within the vessel. This is a necessary simplification, as such a study is impossible to carry out using direct, invasive BP measures. However, while there is the possibility of minor auto-regulation effects within the brachial artery, the effect should be very limited. Another limitation of our study stems from the fact that sleep times were defined based on self-reported data from the participants. This issue may introduce some variability in defining the precise asleep intervals. Upright body positions were detected during the asleep measurements, which could have occurred when participants got up to use the toilet or simply elevated their upper body. Moreover, the body position sensor only distinguished between different body postures (e.g., upright, supine, left) without providing specific angle measurements, limiting our ability to make more precise distinctions within the different postures. We observed that hydrostatic pressure in the upright position during the night was lower than during the day, likely due to participants assuming a partially upright posture rather than being fully upright.

This study clearly indicates the need for future research and technological advancements in ABPM. It is crucial to refine our measurement technologies to provide accurate and reliable measures of BP with as little external effects as possible. The demonstration of the large effect size and clinical impact of hydrostatic pressure differences between the cuff and the heart level necessitates research aiming at incorporating hydrostatic pressure adjustments into ABPM.

## Conclusion

We were able to demonstrate major effects of hydrostatic pressure differences between the cuff and the heart level during ABPM. These effects were especially pronounced during nocturnal measurements and closely related to changes in body posture.

On average, the measured arm was above the heart level, leading to a lower hydrostatic pressure at the BP cuff. In consequence, correcting the hydrostatic pressure difference led to increased mean BP levels, again especially pronounced during the night. The correction changed the nocturnal hypertension diagnosis and/or the dipping pattern classification in almost 50% of patients.

Using the detection of axial body postures effectively limits the error induced by hydrostatic pressure differences and can easily be implemented in ABPM measurements.

Our results highlight the need for recognizing the effects of hydrostatic pressure during ABPM and offer a possible explanation for the limited reproducibility of nocturnal dipping classifications and the lower predictive thresholds in ABPM when compared to office BP measurement.

Consequently, there is a strong impetus for future research to explore the potential integration of hydrostatic pressure compensation mechanisms into ABPM. Incorporating these considerations could greatly improve the accuracy of BP measurements, leading to better hypertension diagnosis and management.

## Supplementary information

Supplementary Information
