# Supplementary material for: Blood pressure measurement and nocturnal dipping patterns are heavily affected by body posture through changes in hydrostatic pressure between the arm and the heart
Source: Hypertens Res. 2024 Dec 6;48(3):1144–54. doi: 10.1038/s41440-024-02056-0 (PMC11879860; doi:10.1038/s41440-024-02056-0)
Supplement: Supplementary file 1 — Supplementary Information [file 41440_2024_2056_MOESM1_ESM.docx]

**Supplement Figure 1**

*
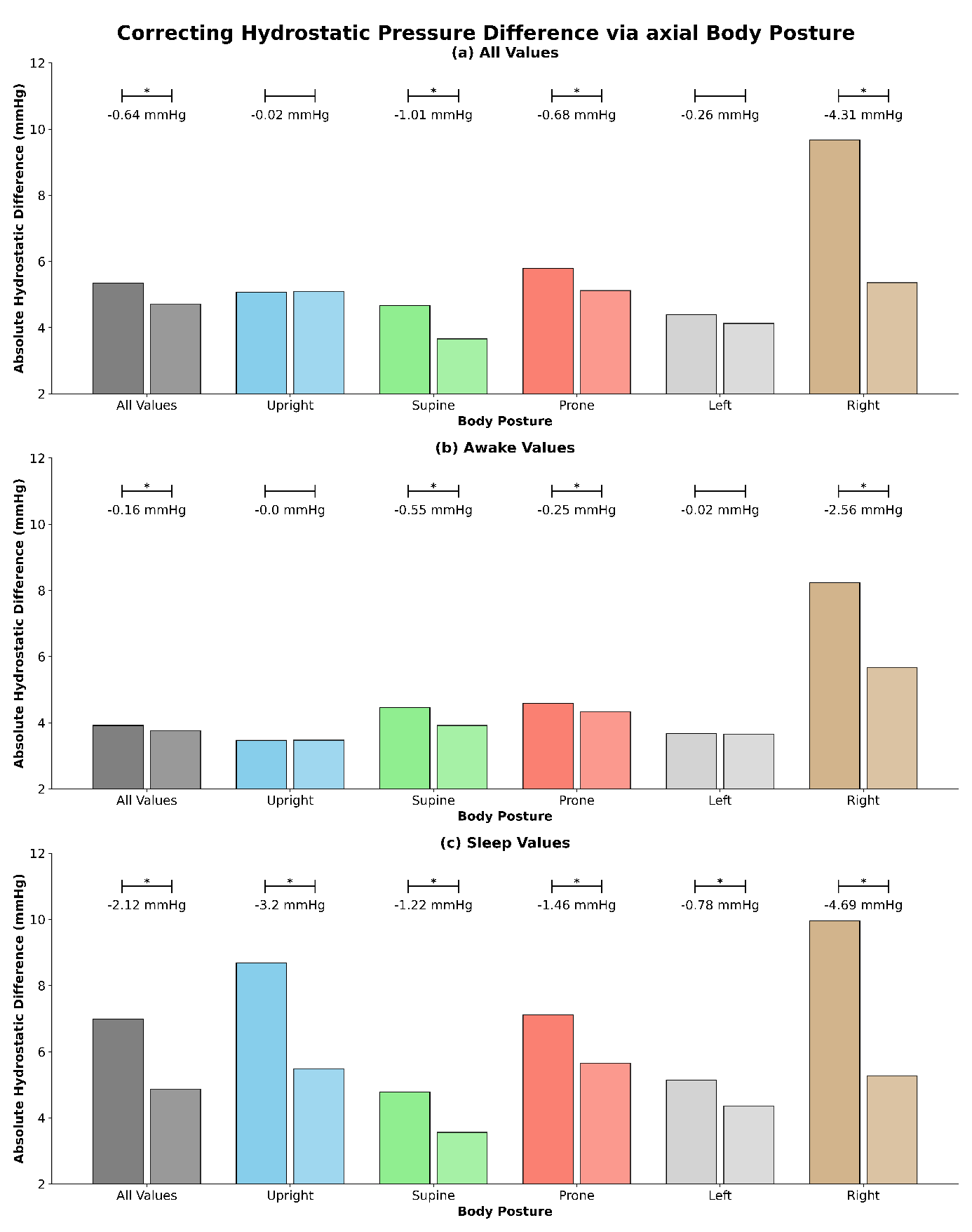
Supplement Figure 1: Hydrostatic Pressure Difference Correction via axial Body Posture: The figure illustrates the reduction in absolute hydrostatic pressure difference between cuff and heart level, when corrected by the mean value for each body posture. The left bars represent the uncorrected absolute difference, the right bars (lighter colour) represent the corrected differences. The three panels depict the effect for all values (a), awake (b) and sleep (c) values. * = p < 0.05*
